# Oral Mucosa Capillaroscopy: A Narrative Review

**DOI:** 10.3390/cancers16223774

**Published:** 2024-11-08

**Authors:** Maria Contaldo

**Affiliations:** Multidisciplinary Department of Medical-Surgical and Dental Specialties, University of Campania Luigi Vanvitelli, Via Luigi de Crecchio, 6, 80138 Naples, Italy; maria.contaldo@unicampania.it

**Keywords:** oral mucosa, oral cancer, oral lichen planus, rheumatic diseases, burning mouth syndrome, Sjogren disease, Behçet’s disease, systemic sclerosis, oral potentially malignant disorders, OPMDs, oral pathology, oral medicine, imaging, non-invasive imaging, capillaroscopy, capillaries, vascularization, inflammation, cancerization

## Abstract

The oral cavity may suffer from diseases and lesions of different natures that can result in changes to the underlying microvasculature. These changes are typically observed during the examination of biopsy samples, but there is still a need to investigate methods for characterizing them in live tissues. Capillaroscopy, a medical imaging technique using polarized light and magnification, has shown promise in providing enhanced imaging of the oral mucosa microvasculature in preliminary studies. The present work proposed to review the literature on what capillaroscopy is, its applications in the imaging of oral mucosa microvasculature, and its diagnostic and prognostic significance in various diseases and conditions. While there is the limited literature available, further research in oral capillaroscopy, particularly in the field of oral oncology, is needed to determine its potential benefits in diagnosing and predicting outcomes for potentially malignant oral disorders and cancers.

## Simple Summary

This article aims to provide a first-of-its-kind summary of the literature on oral capillaroscopy—a non-invasive technique used to study the structure of tiny blood vessels beneath the mouth’s surface in different conditions and lesions. This research has shown that oral capillaroscopy can provide both qualitative and quantitative insights into the health of these microvessels and highlight differences and peculiarities in specific diseases and conditions. This technology has promising applications in oral pathology and oncology, where it can be used to study cancer-related blood vessel growth in real time and monitor chronic inflammatory diseases. Future research may focus on using capillaroscopy to image oral potentially malignant disorders and better understand their microvascular components for diagnostic and prognostic purposes.

## 1. Introduction

Diagnosing mucosal lesions in oral pathology and medicine can be challenging because many diseases and lesions can resemble each other. A clinical examination alone may not be enough to provide a conclusive diagnosis.

While the presence of oral potentially malignant disorders (OPMDs) is a warning sign [1,2], it is not possible to predict if or when they will develop into cancer since the risk of malignant transformation greatly varies among them [3]. Furthermore, the multifactorial risk of malignant transformation is also influenced by genetic, epigenetic, and environmental factors [4,5]. Last, oral cancers may also arise from allegedly healthy oral mucosa without being anticipated by any identifiable precursor lesions [6,7].

Another challenge is managing chronic diseases affecting the oral mucosa [8,9]. These conditions, which may be inflammatory or immune/dysimmune, may persist for several years and require repeated drug treatments. In some cases, diseases like oral lichen planus can even lead to cancer [10].

In all these situations dealing with lesions suspected to be cancerous, as well as in case of chronic oral diseases, strict and long-term follow-up is necessary to establish any responsiveness, worsening, or cancerization, and the most reliable method for diagnosis and assessing any tumoral derailment is still through histopathology on a biopsy specimen [11,12,13]. However, this approach, although necessary, inescapably limits the patient’s compliance and increases the risk of dropping out of the follow-up [14].

In this complex scenario, we welcome any imaging procedures that can non-invasively screen at-risk individuals and monitor their responsiveness to treatments or the progression of chronic and potentially malignant disorders. The goal is to enhance the clinical routine by enabling early diagnosis and ameliorating monitoring over time, which will benefit the patients and their compliance.

For these purposes, the literature has provided numerous examples of non-invasive imaging tools that can assist with clinical decision-making to enhance and refine diagnostic procedures in a clinical setting without causing harm [15,16,17]. It is the case of in vivo confocal microscopy [18,19,20], intraoral high-frequency ultrasound echography [21,22], optical coherence tomography [23,24,25], and tissue autofluorescence [26,27] that allow for optical biopsies at a microscopic resolution similar to conventional histology [18,19,20], measure the depth of tumor invasion [21,22,23,24,25], define the homogeneity or inhomogeneity of a mass [21,22], assess its thickness [21,22,23,24,25], and detect its biochemical or metabolic changes [26,27].

Furthermore, oral diseases and lesions often exhibit specific changes in their subepithelial microvasculature [28]. These changes are typically observed during histopathological assessment of biopsy specimens, but they are still poorly investigated in vivo. Two main imaging tools, narrow-band imaging (NBI) and capillaroscopy, have been reported in the literature to address this. They play a crucial role in evaluating these changes in vivo, thereby enhancing the diagnosis and follow-up of oral diseases.

NBI involves using a standard endoscope with magnification and a white-light source enhanced with green–blue lights to highlight the submucosal blood vessels. The shape and features of these blood vessels can help predict the nature of the lesion [15,29]. NBI is widely used for prognostication and understanding microvascular alterations associated with inflammatory diseases, OPMDs, and malignancy based on capillary density, tortuosity, and branching patterns [29].

On the other hand, capillaroscopy, originally developed for skin application, has been successfully adapted for oral mucosa imaging. This adaptability, combined with its use of polarized light and magnification, allows it to visualize the sub-epithelial capillary bed in the oral mucosa. As a result, it provides detailed information about local microcirculation and vascular patterns [30], similar to NBI. This adaptability not only enhances its versatility but also hints at its potential for further developments and applications in the future.

However, despite the convenience and cost-effectiveness of capillaroscopy devices, which could facilitate their widespread use in clinical settings of dentists and oral pathologists, there is a noticeable lack of comprehensive work on capillaroscopy [31].

The literature on oral mucosa capillaroscopy encompasses an inhomogeneous number of case/control studies that compare the healthy features of oral subepithelial capillaries with those in a series of local and systemic conditions, such as the effects of smoking, the microangiopathies in diabetics, and the oral manifestation of rheumatic diseases.

Surprisingly, despite the potential of capillaroscopy, no comprehensive studies have been conducted to collect and summarize its findings in the context of oral pathologies and lesions. This glaring gap in the literature underscores the urgent need for this review, which will help understand the potential and future directions of oral capillaroscopy.

Hence, the present work aims to review the scientific literature on capillaroscopy applied to the oral mucosa, investigating its clinical applications, outcomes, potential differences in microvasculature observed in various oral mucosal diseases and conditions, and their diagnostic and prognostic significance.

### 1.1. Study of the Microcirculation and Oral Capillaroscopy: The Past and the Present

Professor Friedrich Jung’s recent paper examines the history of microcirculation studies [32]. In the early 1600s, Andrea Cesalpino coined the term “*capillari*”, derived from the Italian “*capelli*”, meaning “hair”. Approximately 60 years later, Marcello Malpighi [33] and Antoni van Leeuwenhoek [34] described microvessel anatomy in animals for the first time. Hermann Boerhave conducted the first in vivo imaging of human microvessels, focusing on the bulbar conjunctiva, revealing key insights about slowed blood flow and the presence of erythrocytes and platelets [35]. Jung also pointed out that intravital microscopy could not use vessel classification based on wall structure, such as the lack of capability to identify muscle cells at the arteriole–capillary transition as in histology. Instead, in vivo capillaroscopy classifies vessels based on diameter, branching, length, density, and shape [32].

Based on these findings, the in vivo imaging of capillaries, known as “capillaroscopy”, is a technique that focuses on elucidating the morphological features of microvessels in easily accessible human districts, like the nail fold. First demonstrated in 1965 by Unna et al., this method allowed for visualization of microvessels after oiling the fingernails [36]. Since the introduction of video and computer technology in 1973, capillaroscopy has significantly advanced, enabling detailed qualitative and quantitative analysis of capillary features linked to various skin and systemic diseases [37,38]. Today, high-definition videocapillaroscopes combined with image analysis enhance diagnostic capabilities by providing precise measurements of microvessels with diagnostic support purposes.

### 1.2. Capillaroscopy Applied to Oral Mucosa: Technical Characteristics and Operating Instructions

A capillaroscope is a standard light microscope primarily used to examine nailfold capillaries and skin areas. When it is equipped with a video camera and a recording system, it is referred to as a videocapillaroscope [30]. Modern videocapillaroscopes are also defined as “video–biomicroscopes in epiluminescence with immersion and polarized light”. These devices typically consist of the following [39]:

- an optical probe with a cold polarized light source, which illuminates the area of interest and highlights the subepithelial vessels, featuring adjustable intensity;
- a video–optical terminal connected to the probe and processing unit, which includes a video sensor and supports variable magnification optics (up to 1000×);
- a processing unit with integrated software for image digitization, storage, and analysis of capillary parameters such as length and diameter, as well as the calculation of capillary density;
- a color monitor that displays capillaroscopic images in high resolution (up to 420,000 pixels).

**Figure 1:**
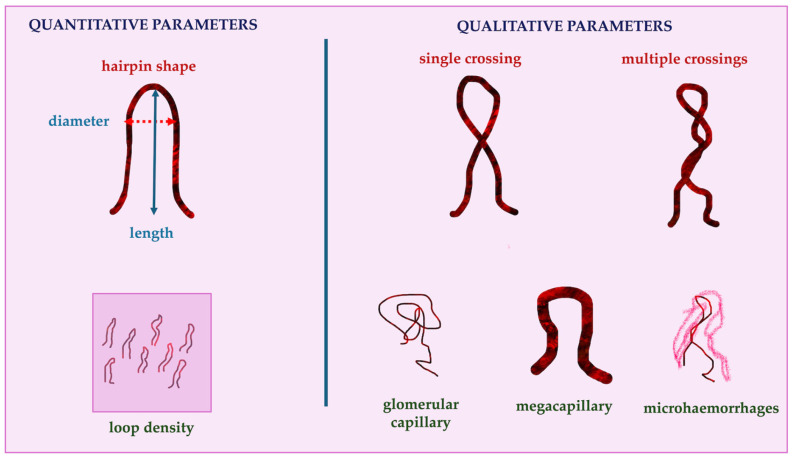
A schematic representation of the main quantitative and qualitative oral capillary features.

The literature identifies two main types of variables used to describe and evaluate oral capillary vessels. These variables are dichotomized into quantitative (parametric data), which accurately measure and count using software integrated with the capillaroscope, and qualitative (nonparametric) parameters (Figure 1), as follows [40,41,42]:

1. Quantitative parameters:
  - Loop length (normal range, 150–500 μm), which also depends on the loop orientation: the more parallel to the surface, the longer the capillary appears under capillaroscopic examination;
  - Loop density (the number of capillaries counted in a field of view, normal range, 12–20 capillaries/mm^2^);
  - Loop diameter (mean normal range, varying per intraoral site, 9–30 μm);
2. Qualitative parameters
  - Visibility of the loops, indicating how easy or difficult it is to identify the capillaries during the in vivo imaging;
  - Loop orientation, which is related to the mucosal surface and can be categorized as parallel, perpendicular, or a combination of both;
  - Loop tortuosity, which is classified based on the number of crossings or twists seen in a 1 mm^2^ area and is scored on a four-point system:
    (0) not crossing/hairpin shape/reverse “U” shape;
    (1) one crossing/single twist;
    (2) multiple crossings/multiple twists/corkscrew shape;
    (3) complete distortion/ball-like aspect;
  - microhemorrhages, present/absent;
  - capillary monstrosities (megacapillaries, glomerular capillaries).

## 2. Research Methods

The search was conducted by investigating two different databases (PubMed and Scopus) with the following keywords and their synonyms, combined with the Boolean operators (AND, OR): capillaroscopy; capillaroscopic; oral medicine; oral mucosa; mouth; oral cavity; oral oncology; oral carcinoma; oral squamous cell carcinoma; oral cancer; oral potentially malignant disorders; lip cancer; actinic cheilitis; leukoplakia; erythroplakia; oral lichen planus; oral ulcers; oral submucous fibrosis; smokers; lupus erythematosus; candidiasis; oral lichenoid lesions; oral lichen planus; rheumatic diseases; inflammation.

The search was conducted without any restrictions.

The eligible articles were original studies on humans that underwent oral mucosa capillaroscopy with available full text and published in English. Conversely, studies on animal/cellular models, those written in languages different from English, and those with full-text unavailable were excluded. All the reviews, letters, proceedings, meeting abstracts, and editorials were not considered from qualitative analysis but read for searching, eventually cross-referenced eligible articles. Data from the eligible papers were organized in tables, summarizing the references, the aims, the methods, the parameters considered, the main findings, and the conclusions for any study.

## 3. Results

The literature is summarized in Table 1.

The main condition-related features are reported in Table 2.

### 3.1. Capillaroscopy Features of Healthy Oral Mucosa

Scardina et al. have significantly defined the baseline capillaroscopy features in healthy oral mucosa. They systematically imaged capillaries from various sites of the oral mucosa using a videocapillaroscope with a magnification range between 50× and 200×, with a preference for 200× [41,43,44]. Their research primarily focused on parametric and nonparametric features of oral capillaries in healthy individuals and laid the foundations for subsequent studies on oral capillaroscopy in various diseases and conditions.

In the mucosa of the lip and cheek, capillary loops are regularly aligned predominantly parallel to each other, with a mean length of 150–500 μm, an inverted U-shape, a defined hairpin shape, and a relatively regular diameter varying between 9 and 30 μm, with a homogeneous density of 12–20 capillary loops/mm^2^ [41,43]. Conversely, at the gingival margin, the capillaries run perpendicular to the surface, appearing like dots or commas [44], while their course is mixed at the retrocommisures and ventral tongue [45].

These observations were consistent with conventional histology, showcasing perfect analogies in the observation of in vitro and in vivo microcirculation, with minimal error [43,72,73]. Rare reddish stains indicating traumatic microhemorrhages were also occasionally reported [45]

The ventral tongue, due to its thinner epithelial layers, produced higher-quality images, making examination easier, and the examination performance was easier in the covering mucosa of the lower lip and cheek compared to gingival levels [41,45]. Capillary loop density was the easiest to calculate at the gingiva, where the capillary course was predominantly perpendicular to the surface [45].

It was noted that oral capillary parameters could vary according to age and sex [45]. On average, capillary loop density and length are significantly higher in women than men. Additionally, capillary density tends to increase with age, with women showing a higher increase in the fifth decade (menopausal period) and men after the sixth decade, reaching similar densities by the eighth decade [45]. Menopausal women showed a shorter loop diameter, increased lip vessel tortuosity, and decreased gingival capillary density compared to premenopausal women [39].

An ethnic comparison study found no significant differences in lingual capillary features between Chinese and Flemish individuals [74].

### 3.2. Capillaroscopic Changes in Autoimmune Rheumatic Diseases

Rheumatic diseases encompass a variety of disorders that primarily impact the joints and multiple systems in the body. They result from immune system issues, inflammation, infections, or gradual deterioration of joints, muscles, and bones [75,76]. Abnormalities in the microvasculature are common. Consequently, conventional nail fold capillaroscopy is used to identify specific diagnostic signs of different rheumatic diseases. Building on this groundwork, some authors have employed oral capillaroscopy to examine the oral microvasculature in patients with various rheumatic diseases, yielding notable results.

### 3.3. Oral Capillaroscopy in Systemic Sclerosis (SyS)

Systemic sclerosis is a rare, chronic autoimmune disease that impacts the body’s connective tissues, leading to degeneration and scarring in the skin, joints, and internal organs, as well as causing abnormalities in blood vessels [67]. As abnormal nail fold capillaries are included in the diagnostic criteria, some researchers have sought to investigate capillaries in the oral mucosa to determine if similar or distinctive signs also appear in this location.

All studies conducted on this group of patients have identified significant differences in specific oral capillaroscopic parameters compared to healthy controls. The first study to utilize capillaroscopy on the oral mucosa was conducted in 1993 by Grassi et al. In this study, 13 women with systemic sclerosis and 11 healthy controls were examined to assess microvascular changes in the lips using a stereomicroscope and computer-aided morphometric analysis [46]. This study found that 92% of SyS patients had abnormal architectural disruptions in the capillary network of the lips, and the capillary loops were significantly shorter, while the density was significantly higher compared to the control group [46].

In 2005, Scardina et al. analyzed gingival microcirculatory abnormalities in patients with systemic sclerosis [47]. They reported a significantly lower capillary density, defined as “desertification”, and enlarged capillary calibers.

Conversely, in a study on rheumatoid arthritis [49], the same authors reported that the capillary loop diameter was lower in these subjects than in healthy subjects, while capillary density increased.

Recently, Antonacci et al. [48] described orofacial manifestations and some lip and gingival capillaroscopic features from 25 patients with systemic sclerosis compared with 15 healthy controls. The patients, compared with controls, characteristically reported more frequent microhemorrhages in both gingiva and lips, tortuosity in more than half of the patients, and significantly more severe difficulty in visualizing the vessels. These findings correlated with the presence of scleroderma.

#### 3.3.1. Oral Capillaroscopy in Sjögren’s Syndrome (SS) and Hashimoto’s Thyroiditis (HT)

*Sjogren’s Syndrome* is a chronic antibody-related autoimmune rheumatic disease characterized by a progressive lymphocytic infiltration of exocrine glands, especially salivary and lachrymal ones, leading to xerostomia, parotid gland enlargement, and xerophthalmia [70,77].

The capillaroscopy of the labial mucosa in patients with Sjögren’s Syndrome revealed that capillary loops were significantly more twisted, and the average capillary diameter was significantly wider and enlarged compared to healthy controls [50]. The density of capillaries was significantly increased, while the length was significantly shorter [50]. At the level of the interdental papilla, SS patients showed significantly higher capillary diameter, density, and tortuosity than healthy controls, without any significant differences related to loop length [51].

Similarly, a case-control study examining the oral microvascular features in subjects with Hashimoto’s thyroiditis (HT) and an autoimmune disorder of the thyroid gland [52] found significantly higher capillary density, significantly lower loop caliber, and that the vascular shape significantly differed, being mainly tortuous and completely distorted in 67% of HT patients [52].

#### 3.3.2. Oral Capillaroscopy in Behçet’s Disease (BD)

Behçet’s disease is an auto-inflammatory multisystemic vasculitis that presents with oral symptoms such as aphthous-like oral ulcers [78].

In 2021, Demirbaş et al. [53] found that oral mucosal capillaroscopy could potentially be used to indicate microvascular damage in sixty BD patients with oral aphthae. Through oral capillaroscopy examination, it was observed that BD patients, compared to healthy controls, exhibited significantly higher frequencies of irregular vessels, glomerular vessels, microhemorrhages, megacapillaries, and tortuous vessels. Furthermore, these oral mucosa capillaroscopic findings were found to be correlated with mucocutaneous, systemic, and vascular findings, disease duration, and clinical severity. Specifically, microhemorrhages, glomerular vessels, and megacapillaries were significantly more frequent in patients reporting erythema nodosum, superficial thrombophlebitis, and HLA B51-positivity, while irregular capillaries strongly correlated with erythema nodosum and acneiform rash. It was also noted that irregular capillaries, microhemorrhages, glomerular vessels, megacapillaries, and tortuous vessels increase in frequency with the severity of the disease [53].

### 3.4. Capillaroscopic Changes in Diabetes

Type 2 diabetes mellitus (T2DM) is a chronic metabolic disorder characterized by persistent, prolonged high blood sugar levels that can damage the microvasculature, ultimately causing diabetic microangiopathy and neuropathy of various organs, including the limbs and the periodontal tissues [71,79,80].

Several studies have utilized oral capillaroscopy to examine the characteristics of the oral mucosa microcirculation in individuals with diabetes and the impact of disease duration and treatments on oral microangiopathy [42,54,55].

People with diabetes exhibited significantly increased capillary length and diameter compared to healthy controls [54]. In contrast, capillary density was notably lower in the labial, buccal, and lingual mucosa [54] in relation to the duration of the disease and treatment [42].

Conversely, individuals with diabetes had higher average gingival capillary density compared to healthy controls, regardless of disease duration and treatment [42,55], including pregnant women with gestational diabetes compared to non-pregnant healthy women [57].

When individuals with diabetes experience neuropathic or ischemic foot lesions, they have been found to have a significant reduction in capillary density (approximately 59–78%) compared to both healthy individuals and those with uncomplicated diabetes [56]. Additionally, individuals with foot complications due to diabetes have reported a significant decrease in the average length of their capillaries (approximately −10%), while those with diabetes but without foot complications have shown an average increase of about +69% [56]. Furthermore, a recurring sun-like capillaroscopic pattern has been observed in neuropathic foot diabetics, with capillaries arranged radially around an avascular area [56].

### 3.5. Capillaroscopic Changes in Burning Mouth Syndrome (BMS)

BMS is an idiopathic chronic pain disorder characterized by a constant burning sensation in the mouth without any identifiable cause. It can affect various parts of the oral mucosa and may also be accompanied by xerostomia and taste changes, with controversial findings about microscopic and biochemical changes in the oral mucosa [68,81,82,83].

In a 2008 study, researchers examined the oral microvasculature in BMS patients using capillaroscopy [58]. They found minor variations in loop length but observed a significant increase in loop calibers in the lips of BMS patients. Additionally, the density of loops was higher in the gingiva of BMS patients [58]. Qualitatively, BMS patients showed a higher degree of capillary loop tortuosity and a distinctive pattern of curved, branched, and dilated capillary loops in the lips and tongue [58].

### 3.6. Capillaroscopic Changes in Oral Lichen Planus (OLP)

Lichen planus is a chronic inflammatory disease with still uncertain etiology that can affect the skin and/or mucous membrane. It is characterized by chronic T-cell-mediated immunological dysfunction and reactive, regenerative, or reparative epithelial changes [84]. When it affects the oral mucosa, oral lichen planus (OLP) may present with reticular, erosive, or atrophic patterns. Diagnosis should consider both clinical and histopathological criteria [84]. In addition to specific epithelial changes, narrow-band imaging (NBI) has reported abnormalities in the oral microvasculature, mainly elongated and tortuous submucosal capillary loops [29,69].

The capillaroscopy of the oral lichen planus (OLP) affecting the cheek mucosa showed that the capillary parameters might vary significantly according to the clinical pattern [59]. Compared with healthy controls, only the erosive and atrophic forms, but not the reticular ones, exhibited significantly increased loop density (four-fold higher than controls [62]), significantly larger diameters, and a greater presence of crossings and tortuosity. However, the loop length did not significantly differ in any condition compared with healthy individuals. Among the three clinical patterns, atrophic OLP reported the highest capillary density, loop diameter, and tortuosity and exhibited more branched and tortuous loops under capillaroscopy and histological sections [59,62]. The significantly increased loop density was also confirmed in the tongue’s OLP, and it was higher in subjects with histological basal membrane disruption [60,61].

### 3.7. Capillaroscopic Changes in Smokers

Among the smoke effects on oral mucosa, other than the increased risk of cancerization, the early and persistent onset of chronic inflammation is responsible for periodontal diseases and other local conditions [5,85]. A series of studies investigated the use of oral capillaroscopy to define in vivo the morphologic changes in the microcirculation induced by chronic smoking habit.

Studies have shown that smokers had smaller capillary loop diameters, higher capillary density [65], and more tortuous capillary loops [64] compared to nonsmokers. These changes are directly correlated with cumulative smoking habits over the years, indicating the impact of smoking on microcirculation [40]. Additionally, smokers may experience microaneurysms and microhemorrhages in 20–66% of cases [40,63].

Even after quitting smoking, some vascular alterations may persist [66]. Lip capillary density increases in smokers [64] and doubles in ex-smokers [66] compared to nonsmokers. However, ex-smokers also have shorter capillary loops compared to smokers and nonsmokers [66].

## 4. Discussion

The present work aimed to highlight the current state of knowledge, experiences, successes, and failures of oral capillaroscopy in studying and detecting changes in the oral submucosal microvasculature for diagnostic and prognostic purposes.

From the analysis of the scientific literature, it is evident that there is a limited and uneven number of studies focusing on oral capillaroscopy to examine changes in oral microvasculature across various conditions, primarily autoimmune rheumatic diseases, diabetes, oral lichen planus, and among smokers.

All studies confirm that oral capillaroscopy is a valuable tool for observing and quantitatively measuring common microvascular changes. These changes align with traditional histology, establishing oral capillaroscopy as a reliable method for studying and monitoring oral diseases and systemic conditions affecting oral microvasculature. Consequently, oral capillaroscopy provides indirect diagnostic and prognostic indicators for various conditions [42].

Furthermore, the capillary features observed in the oral mucosa do not always correspond with those from other sites. For example, patients with systemic sclerosis exhibit unique capillary characteristics in the oral cavity that differ from findings in the nail fold [46]. This suggests a need for a distinct and specific classification system for oral capillaroscopic parameters, as the findings in the oral cavity may differ from those observed in other areas, such as the nail bed.

Mean ranges for parametrical variables have been identified in healthy subjects, although these can be significantly influenced by factors such as sex, age, and, in women, menopause, potentially due to hormonal influences and subclinical inflammatory states [86].

The visibility of capillary loops and the effectiveness of examinations are generally better in healthy conditions, especially at the lower lip and gingiva. However, for smokers, this becomes more complex due to increased keratinization in the epithelium, which results from continuous thermal and mechanical stimuli.

In the rheumatic disease group, frequent changes observed included an increase in capillary loop density, as expected in vasculitis and neoangiogenic diseases. Conversely, loop length can either increase or decrease variably compared to healthy individuals, depending on the specific diseases.

The literature on oral capillaroscopy in diabetic patients indicates a significant increase in loop density and length, along with a notable decrease in loop diameters across all intraoral sites assessed, which can be attributed to the disease and its treatment. In cases of oral lichen planus, there were significant increases in both loop density and diameter. Smokers tended to exhibit a decrease in loop length and diameter alongside an increase in capillary density, often with occurrences of microaneurysms and microhemorrhages.

Tortuosity and abnormal shapes were commonly observed in patients with burning mouth syndrome, oral lichen planus, smokers, and those with rheumatic diseases, potentially indicating early signs of these pathologies. Some specific diseases were associated with unique capillary patterns, such as the sun-like capillaroscopic pattern seen in neuropathic diabetic patients [56] and the curved, branched, and dilated capillary loops found in patients with burning mouth syndrome [58]. Additionally, abnormalities in gingival microcirculation in systemic sclerosis were characterized as “desertification” [47]. In Behçet’s disease, changes in oral mucosal capillaroscopy correlated with various clinical findings, disease duration, and severity [53].

A single study described changes in oral microcirculation using an oral capillaroscopic approach in 25 patients undergoing chemotherapy for head and neck cancers, reporting a statistically significant increase in loop diameter and tortuosity, along with a distinct “wound-up ball” appearance [87].

Few studies have explored oral microcirculation in dentistry concerning endodontic and periodontal features under specific conditions. For instance, Lira-Junior et al. employed a handheld videomicroscope to visualize in vivo microcirculation in patients with severe periodontitis, revealing significant endothelial and microvascular dysfunctions [88].

In endodontics, transient effects on gingival microcirculation were observed in relation to using heated instruments for root canal obturation [89]. Oral capillaroscopy revealed that elevated temperatures in the dental canal caused visible changes in the vasculature of adjacent sites; however, these alterations diminished and completely healed within seven days [89].

Finally, concerning oral wound healing and neoangiogenesis, a study involving 20 subjects who underwent punch biopsies for benign neoformations of the cheek, lip, and tongue noted a significant increase in capillary density over time, while loop length initially decreased after two days, but returned to its original length after seven days [90].

The present literature analysis revealed a significant limitation of current methods due to the ergonomics of the devices, which must maintain direct contact with the mucosa being examined. As a result, oral capillaroscopy is not currently able to image the submucosal microvessels in certain intraoral areas, such as the hard and soft palate, the retromolar trigonous region, and the lingual root.

However, Bastos et al. recently proposed a prototype device called “Real-Time Optical Vascular Imaging (RTOVI)” that aims to address this limitation. RTOVI is a videocapillaroscope specifically designed for intraoral examinations [91,92]. It features a green ultra-narrow light, like narrow-band imaging (NBI), and a cold xenon light source. This device acts as a contact endoscope and has been reported by its authors to successfully access challenging areas within the oral cavity. However, preliminary studies conducted by the authors focused on gingival sites in healthy volunteers.

Another critical area that deserves attention is the application of oral capillaroscopy to study the microvasculature in oral carcinoma. Currently, existing research on oral cancers and precancerous microvascular patterns relies primarily on NBI technology, which can differentiate between cancerous and noncancerous tissues, as well as determine the stage of the disease [69,93,94,95,96]. However, there is a clear need for further investigation in this area. Some researchers have begun to explore the potential of optical coherence tomography (OCT) angiographic images [97], but much work remains to be carried out in this promising field.

## 5. Conclusions

Although existing studies on oral capillaroscopy demonstrated the technique’s ability to visualize, quantify, and objectively assess specific parameters being both user-friendly and cost-effective, they remain at a preliminary stage that still requires further standardization and improvements.

The main limitations of this tool include low specificity, high variability in what is considered “normal”, potential challenges with capillary visibility, and significant reliance on the operator’s experience and interpretation. Additionally, a critical limitation is the ergonomics of videocapillaroscopes, which restrict access to certain intraoral regions, thereby limiting the study of diseases and lesions in these areas.

Given that oral capillaroscopy could serve as a complementary tool for diagnosis and follow-up in oral pathology, several strategies are desirable. First, it is essential to implement knowledge about the capillary patterns of pathologies and oral conditions that have already been investigated, such as oral lichen planus and burning mouth syndrome, to consolidate and standardize diagnostic and prognostic outcomes. Second, the application field should be broadened by evaluating various groups of oral potentially malignant disorders (OPMDs) and oral carcinomas at different stages and degrees. This will help establish the true potential of oral capillaroscopy in distinguishing the microvascular features specific to each condition.

Last, as we enter an era of increasing digital assistance in medical procedures, the future of oral capillaroscopy appears promising. An ideal oral capillaroscopy examination will benefit from the development of a computerized support system and AI-assisted image analysis [98]. Such advancements have the potential to detect patterns in complex data and surpass human capabilities and limitations. A first step in this direction has been taken by Taormina et al., who recently reported the development of a computerized support system to stabilize and standardize the images collected through capillaroscopy, making them suitable for automatic segmentation for deep-learning procedures [99]. Furthermore, texture analysis tools have shown promise in distinguishing the nature of different lesions through mathematical and statistical analyses of pixel patterns or fractal dimensions of digital images [98,100,101,102].

## Figures and Tables

**Table 1:** The main literature on oral capillaroscopy investigations.

First Author (Year) Country Journal	Type of Study	Clinical Condition	Oral Site and Subjects	Device Used #	Capillary Loop Parameters Considered	Main Findings	Conclusions
Scardina (2003) Italy Italian J Embriol [41]	cohort	Healthy	lip, gingiva 100 healthy subjects	A	visibility; course; caliber; length; density; tortuosity; microhemorrhages	Visibility was easier in lip than gingiva. Lip: Capillary loops run mainly parallel (horse stirrup/hairpin shape). Gingiva: Capillary loops run mainly perpendicular (dot/comma shape) to the surface of gingiva. Rare microhemorrhages with the aspect of reddish stains, probably caused by microtraumas. Age and sex influence capillary density and length. Menopausal women reported decrease in capillary diameter, increase in tortuosity, and decrease in density.	Oral capillaroscopy is possible and feasible on lips, gingiva, and tongue. Age, sex, and menopausal state influence some quantitative oral capillary loop parameters.
Scardina (2005) Italy Reumatismo [43]	cohort		dorsal tongue 10 M, 10 F; mean age 42 yr				
Scardina (2007) Italy Ann Histol Embryol [44]	cohort		interdental papilla (10 M, 10 F; mean age 43 yr)				
Scardina (2009) Italy Ann Anat [45]	cohort		lip, cheek, gingiva, ventral tongue 12 M, 33 F; mean age 60 yr				
Scardina (2012) Italy Gerodontology [39]	case/ control		lip, gingiva 27 post-menopausal women 27 pre-menopause women				
Grassi (1993) Italy Ann Rheum Dis [46]	case/ control	Systemic Sclerosis (SyS)	lip 13 F with SyS 11 healthy F	B	length; diameter; density; megacapillaries tortuosity; microhemorrhages	Oral capillaries of SyS subjects exhibited: frequent abnormalities; more tortuosity; greater diameter; shorter length; lower density; frequent microhemorrhages.	Oral capillaroscopy in patients with SyS showed significant microvascular changes compared with healthy controls. Capillary alterations in patients with SyS are not limited to the nailfold bed but also occur in lip and gingival microcirculation.
Scardina, (2005) Italy J Periodontol [47]	case/ control		gingiva 15 Sys/15 healthy 6 M, 24 F; mean age 61 yr	A			
Antonacci (2024) Italy Diagnostics [48]	case/ control		lip, gingiva 25 Sys/15 healthy Sex not reported; mean age 50 years	n.s.			
Scardina (2006) Italy Ann Anat [49]	case/ control	Rheumatoid Arthritis (RA)	lip 30 RA/30 healthy 20 M, 40 F; mean age 62 yr	A	visibility; course; caliber; density; tortuosity;	RA subjects exhibited longer oral capillaries but reduced in caliber compared with controls.	Oral capillary changes could be extremely important in the diagnosis of suspected RA.
Scardina (2009) Italy Ann Anat [50]	case/ control	Sjögren Syndrome (SS)	lip 20 SS/20 healthy 6 M, 34 F; mean age 58 yr	A	visibility; course; caliber; microhemorrhages	Oral capillaries of SS subjects exhibited: evident alterations; reduced diameter; greater density; more tortuosity.	SS subjects reported oral capillary alterations, which could help to complete the diagnosis.
Scardina (2009) Italy Vasc Risk Factor [51]	case/ control		gingiva 25 SS/25 healthy 10 M, 40 F; mean age 53 yr				
Scardina (2008) Italy Ann Anat [52]	case/ control	Hashimoto’s Thyroiditis (HT)	gingiva 15 HT/15 healthy 10 M, 20 F; mean age 23 yr	A	visibility; course; caliber; density	Gingival capillaries of HT subjects exhibited: reduced caliber; greater density; more tortuosity.	HT subjects exhibited gingival microvascular alterations.
Demirbaş (2021) Turkey Dermat Pract Concept [53]	case/ control	Behçet Disease (BD)	lip 60 BD/60 healthy 65 M, 55 F; mean age 35 yr	C	microhemorrhages; glomerular vessels; megacapillaries; tortuosity; irregularities;	BD patients reported significantly higher frequencies of the following: irregular capillaries; microhemorrhages; glomerular vessels; megacapillaries; tortuous vessels.	Oral capillary alterations in BD subjects aligned proportionally with the disease duration, severity, and vascular complications.
Scardina (2011) Italy Panminerva Med [54]	case/ control	Diabetes	lip 23 diabetics/23 healthy 19 M, 27 F; mean age 63 yr	A	length; caliber; density; tortuosity; microhemorrhages	Gingival capillaries of diabetic subjects exhibited the following: higher density; higher length; higher diameter. Lip and tongue capillaries of diabetic subjects exhibited the following: reduced density; higher length; higher diameter. Oral capillaries of diabetics with neuropathic foot peculiarly and significantly exhibited the following: reduced density; reduced length; increased tortuosity; a recurrent capillaroscopic sun-like pattern, with capillaries arranged radially around an avascular area.	Diabetics exhibited significant alterations of the oral capillaries, differing by oral site and proportional to disease duration. Oral capillaroscopy is a potential diagnostic adjunct in the early andsubclinical identification of microangiopathic damage in patients with diabetic foot.
Scardina (2012) Italy Med Sci Monit [55]	case/ control		gingiva 40 diabetics/40 healthy 35 M, 45 F; mean age 63 yr				
Scardina (2017) Italy Med Sci Monitor [42]	case/ control		lip, tongue, cheek, gingiva 60 diabetics/60 healthy 62 M; 58 F mean age 56 yr				
Scardina (2020) Italy J Clin Med [56]	case/ control		Lip, cheek, gingiva45 diabetics/15 healthy male/female ratio: 8/7; mean age 65 yr				
Yilmaz (2021) Turkey Micorvasc Res [57]	case/ control		gingiva 30 gestational diabetes/25 healthy unpregnant controls 55 females; mean age 29 yr				
Scardina (2008) Italy JADA [58]	case/ control	Burning Mouth Syndrome (BMS)	Lip, gingiva, ventral tongue 14 BMS/14 healthy 9 M, 19 F; mean age 60 yr	A	diameter; density; tortuosity	BMS patients reported a statistically significant increase in the oral capillary diameter.	BMS subjects showed vascular differences compared with healthy controls.
Scardina (2007) Italy Oral Surg Oral Med Oral Pathol Oral Radiol Endod [59]	case/ control	Oral Lichen Planus (OLP)	cheeks 20 OLP/20 healthy 17 M, 23 F; mean age 59 yr	A	diameter; density; tortuosity; length;	Oral capillaries of OLP patients exhibited the following: increased density; increased tortuosity; increased diameter; characteristic branched loops.	Oral capillaroscopy allowed for in vivo observation of the angiogenesis in patients suffering from OLP and a consistent correspondence with histology. OLP-associated angiogenesis was interpreted as an increase in capillary density, total vascular caliber, and tortuous, branched loops.
Scardina (2009) Italy Indian J Dent Res [60]	case/ control		tongue 28 OLP/14 healthy 16 M, 26 F; mean age 58 yr				
Scardina (2009) Italy J Oral Sci [61]	case/ control		tongue 10 OLP/10 healthy 2 M, 18 F; mean age 63 yr				
Scardina (2011) Italy Arch. Immunol. Ther. Ex [62]	case/ control in vivo/ in vitro		oral site not specified 30 OLP/30 healthy 14 M, 46 F; mean age 53 yr				
Molino Lova (2002) Italy Am Heart J [40]	case/ control	Smoking	lip 50 smokers/50 nonsmokers 50 M, 50 F; mean age 44 yr	D	length; diameter; density; tortuosity; microaneurysms; microhemorrhages	Oral capillaries of smokers exhibited the following: lower diameter; higher density; more tortuosity; microaneurysms; microhemorrhages.	Oral capillary changes in smokers were proportional to cumulative smoking habits. There was no remission of vascular damage, even 13 years after smoking cessation.
Scardina (2004) Italy It J Anat Embryol [63]	case/ control		lip 35 smokers/35 nonsmokers	A			
Scardina, (2005) Italy Am J Dent [64]	case/ control		gingiva 35 smokers/35 nonsmokers 35 males, 35 females; mean age 44 yr				
Scardina (2005) Italy Odontology [65]	case/ control		tongue 25 smokers/25 nonsmokers 50 M; mean age 55 yr				
Scardina (2019) Italy Med Sci Monitor [66]	case/ control		oral site not specified 25 ex-smokers 25 smokers 25 nonsmokers 36 M; 39 F; age range 18–85 years				

Legend: # devices used: A, videomicroscope with optical probe, with magnification from 10–1000× and integrated software for measures and calibrations (VideoCap, DS Medica, Milan, Italy); standardized magnification: 200×. B, stereomicroscope (Stereo Star Zoom, American Optical); computer-aided system for morphometric analysis (Oculus 300 Frame Grabber, CORECO). C, handheld dermatoscope using polarized light. (DermLite DL4, 3gen Inc., CA, USA). D, videocapillaroscopy with a Videocap + Videocap 5.1 Plus software (Scalar Co., Ltd., Tokyo, Japan); contact optical probe 500× magnification; Image-Pro Plus 4.0 software (Media Cybernetics, Silver Spring, MD, USA). n.s., not significant.

**Table 2:** Main capillaroscopic condition-associated features.

Capillary Features	Oral Site	Healthy Ranges	Clinical Conditions							
			Systemic Sclerosis	Rheumatoid Arthritis	Sjögren’s Syndrome	Hashimoto’s Thyroiditis	Smokers	Diabetes	Oral Lichen Planus	Burning Mouth Syndrome
Lenght (mm)	lip	min 0.186 ± 0.044 max 0.203 ± 0.023	↓ [67]	↑ [47]			↓ unspecified site [63]	↑ in uncomplicated diabetes ↓ in diabetic foot [57]		n.s. [68]
	cheek	min 0.177 ± 0.060 max 0.245 ± 0.05						↑ in uncomplicated diabetes ↓ in diabetic foot [57]	n.s. [69]	
	gingiva	min 0.06 ± 0.09 max 0.26 ± 0.09			↓ [70]			↑ in uncomplicated diabetes ↓ in diabetic foot [57]		
	tongue	min 0.11 ± 0.9 max 0.198 ± 0.031						↑ in uncomplicated diabetes ↓ in diabetic foot [57]	n.s. [60,62]	n.s. [68]
Density (n.capillaries/mm^2^)	lip	min 19.04 ± 3.16 max 21.16 ± 6.54	↑ [67]	↑ [47]			↑ [39,63]	↓ in diabetic foot [57]		n.s. [68]
	cheek	Min 17.43 ± 2.34 Max 21.170 ± 3.900					↑ [63]	↓ in diabetic foot [57]	↑ [69]	
	gingiva	min 15.42 ± 1.7 max 26.74 ± 3.45	↓ [46]		↑ [50,70]	↓ [51]	↑ [63]	↓ in diabetic foot [57] ↑ [54] ↑ [41] ↑ gestational diabetes [55]		↑ [68]
	tongue	min 20.24 ±5.662 max 22.75 ± 4.79					↑ [63,64,65]	↓ in diabetic foot [57]	↑ [60,62]	n.s. [68]
Loop diameter (μm)	lip	min 15.69 ± 2.07 max 24.000 ±0.004	↑ [67]	↓ [47]			↓ [39,63]	↑ [71]	↑ unspecified [59]	↑ [68]
	cheek	min 26.000 ± 0.009 max 29.000 ± 0.007							↑ [69]	
	gingiva	min 9.220 ± 1.44 max 16.66 ± 1.10	↑ [46]			↓ [51]	↓ [63,65]			
	tongue	min 12.50 ± 1.46 max 30.00 ± 0.04							↑ [60,62]	

Legend: ↑, incrase; ↓ decrease; n.s., not significant.
